# Altered Spontaneous Activity in Patients with Persistent Somatoform Pain Disorder Revealed by Regional Homogeneity

**DOI:** 10.1371/journal.pone.0151360

**Published:** 2016-03-15

**Authors:** Tianming Huang, Zhiyong Zhao, Chao Yan, Jing Lu, Xuzhou Li, Chaozheng Tang, Mingxia Fan, Yanli Luo

**Affiliations:** 1 Department of Psychiatry, Shanghai Changning Mental Health Center, Shanghai, China; 2 Shanghai Key Laboratory of Magnetic Resonance, Key Laboratory of Brain Function Genomics, East China Normal University, Shanghai, China; 3 Shanghai Key Laboratory of Brain Functional Genomics (MOE & STCSM), School of Psychology and Cognitive Science, East China Normal University, Shanghai, China; 4 Department of Traditional Chinese Medicine, Tongji Hospital of Tongji University, Shanghai, China; 5 Key Laboratory of Brain Functional Genomics (MOE & STCSM), Institute of Cognitive Neuroscience, School of Psychology and Cognitive Science, East China Normal University, Shanghai, China; 6 Department of Rehabilitation Medicine, Huashan Hospital, Fudan University, Shanghai, China; 7 Department of Psychiatry, Tongji Hospital of Tongji University, Shanghai, China; Institute of Psychology, Chinese Academy of Sciences, CHINA

## Abstract

Persistent somatoform pain disorder (PSPD) is a mental disorder un-associated with any somatic injury and can cause severe somatosensory and emotional impairments in patients. However, so far, the neuro-pathophysiological mechanism of the functional impairments in PSPD is still unclear. The present study assesses the difference in regional spontaneous activity between PSPD and healthy controls (HC) during a resting state, in order to elucidate the neural mechanisms underlying PSPD. Resting-state functional Magnetic Resonance Imaging data were obtained from 13 PSPD patients and 23 age- and gender-matched HC subjects in this study. Kendall’s coefficient of concordance was used to measure regional homogeneity (ReHo), and a two-sample t-test was subsequently performed to investigate the ReHo difference between PSPD and HC. Additionally, the correlations between the mean ReHo of each survived area and the clinical assessments were further analyzed. Compared with the HC group, patients with PSPD exhibited decreased ReHo in the bilateral primary somatosensory cortex, posterior cerebellum, and occipital lobe, while increased ReHo in the prefrontal cortex (PFC) and default mode network (including the medial PFC, right inferior parietal lobe (IPL), and left supramarginal gyrus). In addition, significant positive correlations were found between the mean ReHo of both right IPL and left supramarginal gyrus and participants’ Self-Rating Anxiety Scale (SAS) scores, and between the mean ReHo of the left middle frontal gyrus and Visual Analogue Scale (VAS) scores. Our results suggest that abnormal spontaneous brain activity in specific brain regions during a resting state may be associated with the dysfunctions in pain, memory and emotional processing commonly observed in patients with PSPD. These findings help us to understand the neural mechanisms underlying PSPD and suggest that the ReHo metric could be used as a clinical marker for PSPD.

## Introduction

Persistent somatoform pain disorder (PSPD) is defined as a type of mental disorder that cannot be explained by structural or somatic injury [1]. PSPD patients often suffer from persistent, severe and distressing pain without sufficient explanatory peripheral pathology, and thus it is believed to originate from emotional conflicts or psychosocial problems. Moreover, PSPD is usually non-responsive to a variety of therapies because of PSPD’s unclear pathogenic mechanism. The absence of effective treatment ultimately results in excessive consumption and waste of medical resources in addition to social problems. Obviously, this disease seriously impacts the quality of life of patients and brings a great burden to society [1–5]. It is therefore very urgent for us to investigate the pathogenic mechanisms underlying the functional impairments in the PSPD patient to devise reasonable and effective therapies.

Previous studies have documented two separate pain systems in the human brain having different functional responsibilities [6–9]. One system is the lateral pain system, including the lateral thalamic nuclei, S1 (primary somatosensory cortices), S2 (secondary somatosensory cortices) and other brain regions, and is thought to primarily involve the process of the sensory-discriminative aspects of pain. The other system is the medial pain system. This system includes the medial thalamic nuclei, anterior cingulate cortex (ACC), prefrontal cortex (PFC), insular cortex, and other brain regions. It is believed to be primarily associated with the affective-motivational aspects of pain. An instance of spontaneous pain perception with no physiological basis could be related to an imbalance between the lateral and medial pain systems induced by somatoform pain disorder [10]. Additionally, the Default Mode Network(DMN), including the posterior cingulate, medial prefrontal and inferior parietal cortex, has been demonstrated to be associated with pain-related functional impairments in many studies [8, 11, 12]. To date, the study of PSPD has been scant and limited to the behavior level. For example, Burba et al. (2006) [4], using the Toronto Alexithymia Scale and the Hospital Anxiety and Depression Scale, found that adolescents with PSPD had higher levels of alexithymia and anxiety than healthy adolescent subjects. Schonenberg et al. (2013) [13] utilized a series of animated morph clips and found that while PSPD patients exhibited deficits in mind-reading abilities, their ability to recognize facial expressions was normal. Luo et al. (2014) [2] more recently reported that patients with PSPD received significantly lower scores on the quality of life subscale of the SF-36 relative to the general population, and these differences significantly correlated with pain, depression and anxiety scores, as well. However, these behavior studies cannot reveal the brain mechanism underlying PSPD.

Recently, researchers’ attention has been increasingly attracted to resting-state fMRI, which primarily investigates low-frequency (0.01 Hz-0.08 Hz) fluctuations (LFFs) of blood-oxygenation-level-dependent (BOLD) fMRI signals, and the LFFs are considered to reflect spontaneous neuronal activities [14–17]. This technology is very appropriate for clinical study because it is noninvasive and a complicated task is not need for probing spontaneous brain activity in patients. Regional Homogeneity (ReHo), an analytical method with high test-retest reliability [18], which is calculated using Kendall’s coefficient of concordance (KCC) [19] to measure the similarity between time series of a given voxel and that of its nearest neighbors in a voxel-wise way [20]. This method is suitable and convenient for exploring regional brain activity at rest because it is largely free-parametric (e.g., the choice of seed, thresholds of connectivity and signal-to-noise ratio) and does not require a priori knowledge of the structure or function of the brain [21]. Recent evidence supported that ReHo had biological significance as a functional measure of regional segregation and integration of information processing regarding its cognitive and neuro-developmental aspects [22, 23]. The abnormalities of ReHo within regions of the brain reflect the alterations of synchronization in temporal neural activities [24], suggesting an unbalanced local functionality or a non-compensatory reaction of the whole brain network [25]. This approach has been used to identify specific pathophysiological functional signatures and reveal the neural mechanism underlying dysfunctions across a wide range of diseases including epilepsy [26], Alzheimer’s disease [27], depression [28], and Parkinson’s disease [29]

Several recent studies have used ReHo to investigate the alteration of regional spontaneous activity patterns evoked by pain stimulus or pain disorder. Zhang et al. [30] investigated the changes in resting-state brain activity induced by the low back pain in healthy subjects. They found that, compared with the baseline condition, the scans during experimentally induced low back pain showed increased ReHo in the right medial prefrontal cortex, precuneus, insula, parahippocampal gyrus and cerebellum (posterior lobe), while decreased ReHo in the left S1, ACC, parahippocampal gyrus and right inferior parietal lobule. The authors suggested that the abnormally spontaneous resting-state activity in these brain regions might be associated with pain processing. Yoshino et al. [31] found that patients group with somatoform pain disorder (SPD) exhibited higher ReHo value in the left precentral gyrus compared with health controls (HC), and VAS scores were positively correlated with ReHo in the left precentral gyrus. This finding suggested that the changes in ReHo of the left precentral gyrus might be connected with distinctive neural mechanisms underlying SPD. These studies demonstrate that ReHo is able to reveal the alteration of spontaneous brain activity induced by the pain. However, the difference of the spontaneous brain activity pattern between PSPD and HC across the whole brain is still poorly understood.

To explore the neural mechanism of function impairments uncovered by the previous behavior studies in PSPD patients, the present study investigate the difference in ReHo between PSPD patients and HC and to assess the change in spontaneous brain activity detected in PSPD patients during rest. We hypothesized that there were changes in ReHo (spontaneous activity) of different brain regions in PSPD patients compared with HC. The altered brain activity was mainly found in the brain regions related to the sensation, recognition and emotional dimensions of pain, including some regions of pain systems (e.g., S1, S2, prefrontal cortex) and DMN (e.g. posterior cingulate cortex, medial prefrontal cortex) reported by previous studies [10, 30, 32].

## Materials and Methods

### Participants

Thirteen right-handed patients (eight females, average age: 46.0 ± 14.3 years) who met ICD-10 criteria (International Statistical Classification of Diseases and Related Health Problems, 10th Revision, 1992) for PSPD were consecutively recruited from the psychosomatic outpatient department of Tongji Hospital of Tongji University between May 2012 and May 2015. In addition, 23 HC (eleven females, average age: 46.1 ± 12.7 years) matched in age (p = 0.993, two-sample two-tailed t-test), gender (p = 0.835, Chi-square test), and handedness were recruited via advertisements posted in nearby communities. Inclusion criteria for PSPD patients included: 1) right-hand dominance; 2) ages between 18 and 65 years; 3) duration of clinical pain of at least 6 months; 4) diagnosis of PSPD according to ICD-10 criteria. Exclusion criteria for all subjects included current or past history of any of the following as indicated: 1) presence of pain symptoms due to severe somatic diseases; 2) existence of uncontrolled diseases, such as congestive heart failure, hypertension, cerebrovascular disease, thyropathy; 3) substance or depilatory abuse, such as alcohol and cocaine; 4) presence of mental diseases (such as affective disorder, suicidal depression, anxiety disorder, phobic anxiety disorder, obsessive compulsive disorder, and posttraumatic stress disorder); 5) electroconvulsive therapy within past 4 weeks; 6) current pregnancy; 7) participation in other clinical trials within past 4 weeks; 8) indications as evidenced by magnetic resonance imaging (MRI) of cerebral atrophy diagnosed by a radiologist who visually analyzed the MRI images on the scene. All HC had no chronic/ongoing pain, and did not meet ICD-10 criteria for any psychiatric diagnosis.

This study was approved by the local Ethics Committee of Tongji Hospital of Tongji University (No.141) and conducted in accordance with the Declaration of Helsinki. Written informed consent was obtained for all participants.

### Clinical assessments

The Visual Analogue Scale (VAS) [33] is a psychometric response scale and a measurement instrument for subjective characteristics or attitudes that cannot be directly or objectively measured. When responding to a VAS item, respondents specify their level of agreement with a statement by indicating a position along a continuous line between two end-points. Clinical pain was measured using a 100-mm VAS. The 2 anchors for this VAS were 0 = no pain, and 10 = worst pain imaginable. Patients gave their responses verbally.

The Zung Self-Rating Anxiety Scale (SAS) [34, 35] is a 20-item self-report assessment device built to measure anxiety levels, based on scoring in 4 groups of manifestations: cognitive, autonomic, motor and central nervous system symptoms. The respondent indicates the extent to which each item statement applies to him or her, yielding an overall total raw score ranging from 20 to 80. The raw score is then converted to an "Anxiety Index" (normal range: 20–44; mild to moderate anxiety: 45–59; marked to severe anxiety: 60–74; extreme anxiety: 75–80) to allow for clinical interpretation of one's level of anxiety.

The Zung Self-Rating Depression Scale (SDS) [36] is a short self-administered survey used to quantify the depressed status of a patient. The scale contains 20 items rating the affective, psychological and somatic symptoms associated with depression. There are ten positively worded and ten negatively worded questions. Scores on the test range from 20 through 80. The scores fall into four ranges for interpreting depression severity: normal range: 20–44; mildly repressed: 45–59; moderately depressed: 60–69; severely depressed: ≥70. The demographic characteristics of each patient were displayed in the Table 1.

**Table 1:** Demographic characteristics and clinical assessment of PSPD patients.

ID	Age (yr)	Gender	Handedness	Duration (yr)	VAS	SAS	SDS
01	60	F	R	8	3	28	32
02	43	F	R	1	6	33	39
03	65	F	R	2	4	30	35
04	63	F	R	0.6	10	27	31
05	27	M	R	7	7	51	54
06	43	F	R	5	5	27	28
07	30	M	R	5	5	30	34
08	33	M	R	1	5	55	45
09	27	M	R	6	9	44	43
10	48	F	R	1	3	26	28
11	39	F	R	1	5	37	51
12	62	M	R	4	7	36	30
13	58	F	R	4	5	39	51

### MRI Data acquisition

The MRI images were acquired with a Siemens Trio 3.0 Tesla MRI scanner (Siemens, Erlangen, Germany) at the Shanghai Key Laboratory of Magnetic Resonance, East China Normal University. The Institutional Ethics Committee of East China Normal University (Shanghai, China) approved the study protocol, and all subjects or their guardians discussed and then signed informed written consent. All patients were required to discontinue their pain medications at 1–3 days before their fMRI scan. At the beginning of the fMRI data collection procedure for each participant, the participant’s head was tightly fixed using foam pads to reduce both head movement and scanner noise. Resting-state fMRI data of the whole brain were acquired using an echo-planar imaging (EPI) sequence: 33 axial slices, slice thickness = 4 mm, no gap, matrix = 64×64, repetition time = 2,000 ms, echo time = 30 ms, flip angle = 90°, and field of view = 192 mm×192 mm. T1-weighted images covering the entire brain were obtained in a sagittal orientation employing a magnetization-prepared rapid gradient echo sequence: 192 slices per slab, slice thickness = 0.9 mm, gap = 0.45 mm, repetition time = 2530 ms, echo time = 2.4 ms, inversion time = 1100 ms, field of view = 256 mm×256 mm, flip angle = 7°, and matrix = 256×256. To identify whether the participants had any injury or tumor in their brains, T2-weighted images were also collected using a turbo-spin-echo sequence: 25 axial slices, slice thickness = 4 mm, gap = 1.2 mm, repetition time = 6,000 ms, echo time = 93 ms, field of view = 220 mm×220 mm, flip angle = 120°, and matrix = 320×320. During the functional MRI scanning, all subjects were instructed to keep their eyes closed, relax, and move as little as possible without thinking about anything in particular. Each scan lasted for 8 min and 6 s; however, the first 6 s was consumed by a dummy scan. Thus, a total of 240 image volumes were collected.

### Pre-processing of the functional MRI data

Pre-processing of the fMRI data was performed using Statistical Parametric Mapping (SPM8, http://www.fil.ion.ucl.ac.uk/spm) and the Data Processing Assistant for Resting-State fMRI Data Analysis Toolkit (DPARSF, http://resting-fmri.sourceforge.net). We discarded the first 10 volumes of the data set of each participant to allow for magnetization equilibrium and environment adaptation, thus remaining 230 volumes for further analysis. The images were corrected for the time delay between slices and were co-registered to the first image to correct for rigid-body head movement. Excessive motion was defined as more than 2.5 mm of translation or greater than 2.5 degrees of rotation in any direction. None was excluded due to excessive motion. Also, there were no significant differences in the frame-wise displacement [37] between groups (t = 1.18, p = 0.25). Subsequently, the functional images that underwent motion correction were spatially normalized to the Montreal Neurological Institute (MNI) space using a unified segmentation algorithm [38] and then re-sampled to a 3 mm isotropic voxel using the parameters estimated during unified segmentation.

### ReHo measure and statistical analysis

The ReHo analysis was performed for each subject in normalized images by the Resting-State fMRI Data Analysis Toolkit (REST, http://restfmri.net). First, we used the REST to remove linear trend and conduct temporal band-pass (0.01–0.08 Hz) filtering to reduce low frequency drift and high-frequency respiratory and cardiac noise [39]. To further reduce the influence of confounding factors, a linear regression process was used to remove the effects of other possible sources of artifacts, including six motion parameters, white matter, and cerebrospinal fluid. The ReHo value was then calculated across the whole brain with KCC in a voxel-wise way to assess the similarity of the time series at a given voxel with the time series of its 26 nearest neighbors (the sensitivity of 26 voxels is better than 19 and 7 voxels) [20]:

$$W=\frac{\sum_{i=1,n}{\left(R_{i}\right)}^{2}-n{\left(\bar{R}\right)}^{2}}{\frac{1}{12}{\text{K}}^{2}\left(n^{3}-n\right)},$$

where *R*_*i*_ is the sum rank of the *i*^*th*^ time point,$\bar{R}$ is the mean of the *R*_*i*_′*s*, K is the number of time series corresponding to the spatial voxels contained within a measured cluster (here, K = 27, one given voxel plus the number of its neighbors), n is number of the ranks (here, n = 230 time points), and *i* ranges from 1 to n. Theoretically, ReHo’s advantages are clearly showed from this equation, which integrates noise-filtering operations in both temporal domain (the order-rank filter) and spatial domain (the mean-rank filter). Thus, this metric has high robustness against temporospatial noise and outliers [21]. This equation integrates structural, functional, morphological, geometrical and geographical features for a node, and the neighbor size can be freely adjusted to capture the local connectivity across different spatial scales, such definition also reveals the nature of multimodal and multiscale of ReHo [23]. The KCC value (W) ranging from 0 to 1 was calculated voxel-by-voxel across the whole brain, producing an individual ReHo map for each subject. For standardization purposes, each individual ReHo map was converted to z-values using Fisher’s r-to-z transformation. Subsequently, the standardized ReHo maps for all subjects were smoothed using a Gaussian filter of 6 mm full width at half maximum to reduce noise and residual differences in normalization. Finally, one two-tail one-sample t-test was performed to determine the ReHo distribution in the whole brain within groups, and the other two-tailed two-sample t-test was performed to determine the difference in ReHo between PSPD and HC. Considering that these variances could be scattered across several distributed voxels and induced by noise, a voxel-cluster threshold correction was used in the whole-brain statistics. This correction combined the threshold of contrast maps set at *p*<0.005 for each voxel and a cluster size of at least 74 voxels, which was equal to the corrected threshold of *p*<0.05, as determined by Monte Carlo simulation (parameters were as follows: individual voxel p = 0.005, 1000 simulations, FWHM = 10 mm, with a grey mask) in the REST software.

### Correlation analysis

To determine the relationship between the identified brain regions and clinical assessments. First, we extracted the ReHo index from each PSPD patient in the identified clusters (n = 12) with significant differences against HC subjects. Subsequently, a Spearman correlation (nonparametric) analysis was performed for the ReHo index and VAS, SAS, SDS and duration of illness across all PSPD patients, and p-value < 0.05 was considered to be statistically significant.

## Results

### Participants' characteristics

All 36 participants completed clinical assessment and had an adequate MRI scan. No participant failed in registration of the MRI scan to standard space. Demographic characteristics and scores of clinical assessment of PSPD patients are listed in Table 1. In most cases, participants’ self-reported pain was diffuse. The predominant clinical pain in the patient group was located in the 1) head, neck, and face region (diffuse pain, n = 4); 2) lower back region (lower back pain, n = 1); 3) pelvic region (lower abdomen pain, n = 4); and 4) upper and lower limbs (n = 2). Two patients reported more than one predominant pain location (one patient with lower abdomen pain as well as neck pain, and one patient with pain in the lower limbs along with back pain). There was no significant difference (t = -0.10, p = 0.92) in the VAS score before and after scanning in 13 patients. We considered the patients as severely affected for the diagnostic group of PSPD from a clinical point of view.

### ReHo distribution within groups

**Fig 1:**
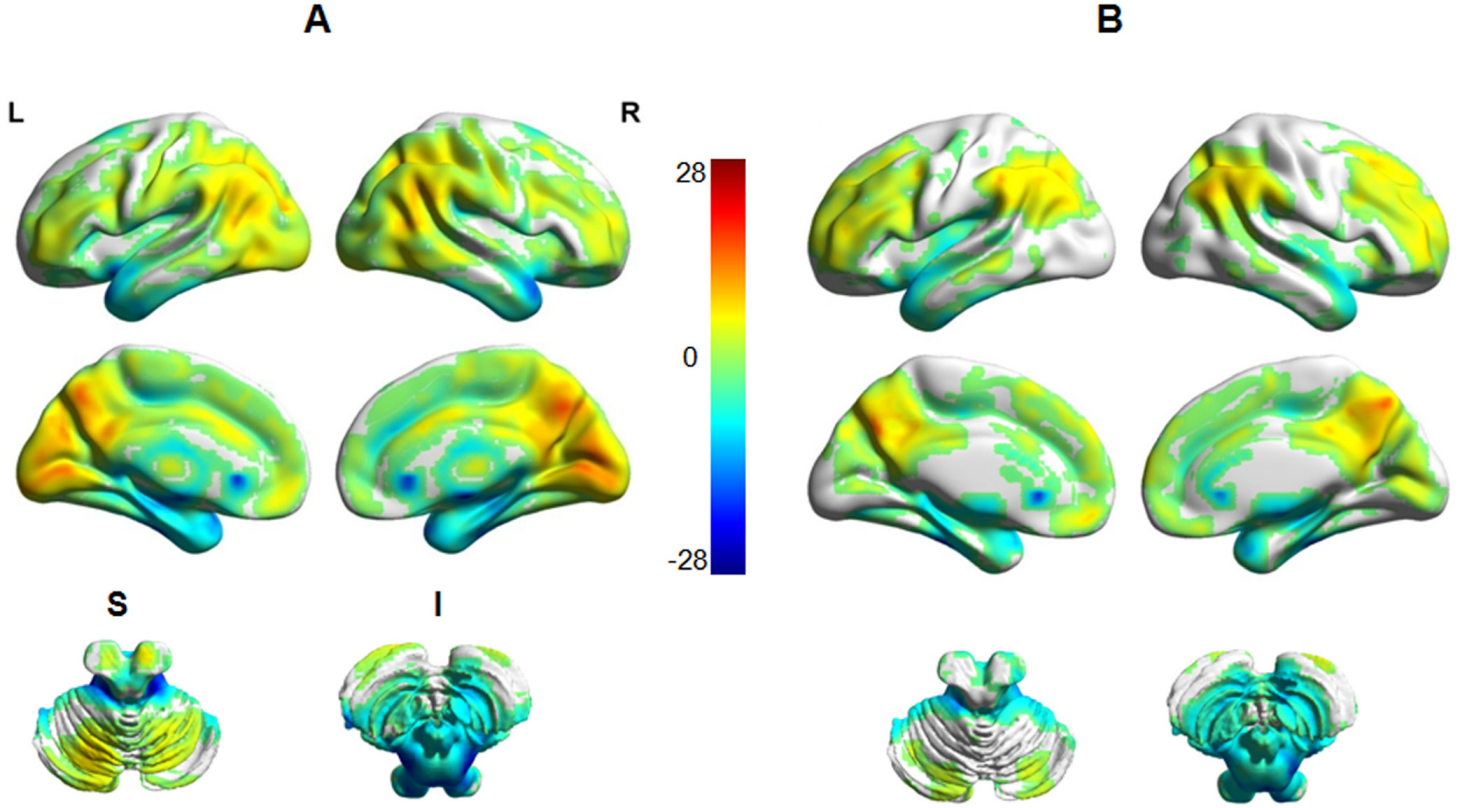
ReHo results for HC and PSPD patients. The ReHo map appears similar distribution between two groups. A: healthy control group; B: PSPD patients. The significance threshold was set at p<0.05 (FDR-corrected) for the ReHo difference between PSPD patients and HC. L: left; R: right; S: superior; I: inferior.

The ReHo results for the HC and PSPD groups are displayed in Fig 1A and 1B, respectively. The primary distribution of ReHo maps for the two groups appear to be similar. Significantly higher ReHo was found mainly in the bilateral sensorimotor cortex, the prefrontal lobe (including bilateral superior frontal gyrus, middle frontal gyrus and inferior frontal gyrus), the default mode network (DMN) (including precuneus\posterior cingulate cortex (PCC), bilateral inferior lateral parietal lobule, and medial prefrontal cortex), cingulate gyrus, occipital lobe and cerebellum. In contrast, significantly lower ReHo was mainly distributed within the bilateral inferior temporal gyrus and brainstem.

### ReHo differences between groups

**Fig 2:**
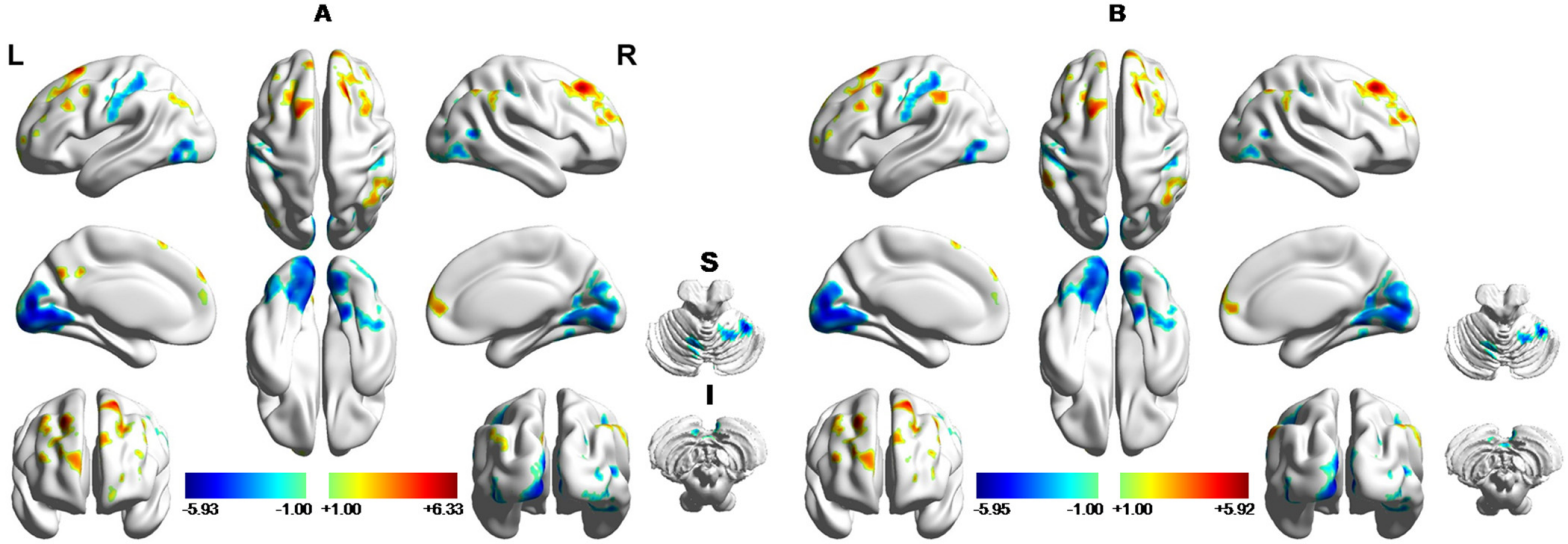
Brain regions showing ReHo difference between PSPD group and healthy controls without (A) and with (B) removing the effect of age, gender and head motions. Red and blue denote higher and lower ReHo in PSPD patients relative to HC subjects, respectively, and the color bars indicate the t-value. The statistical threshold was set at p<0.05 (AlphaSim-corrected).

The two-sample t-test was performed to investigate the ReHo difference between the PSPD and HC groups. Compared to HC, patients with PSPD showed significantly decreased ReHo in the bilateral S1, posterior cerebellum and occipital lobe (Fig 2A and Table 2) while increased ReHo values in the left superior frontal gyrus (SFG), left inferior frontal gyrus (IFG), bilateral middle frontal gyrus (MFG), medial PFC (mPFC), bilateral inferior parietal lobule (IPL) and posterior cingulate gyrus (PCC)/precuneus (Fig 2A). Given the differences in age and gender and the effect of head motion on results, a two-sample t-test was also performed with removing the effect of age, gender and head motion (P<0.05, AlphaSim-corrected). Comparing with the results without removing the covariates (age, gender and head motion), a new cluster emerged at left supramarginal gyrus while left IPL and PCC/precuneus became non-significant in the regions with increased ReHo values after eliminating these covariates (Table 3). The present study primarily focused on the results with removing the covariates (Fig 2B).

**Table 2:** Regions showing significantly lower ReHo in PSPD group than HC.

Regions	BA	MNI coordinates			Cluster	t
		X	Y	Z		
right postcentral gyrus(S1)	2/3	48	-21	36	74	-3.47
left postcentral gyrus(S1)	1/2/3	-48	-24	36	190	-4.28
right occipital lobe	17/18/19	1	-84	1	103	-5.60
left occipital lobe	17/18/19	-6	-87	9	106	-5.92
right posterior cerebellum		6	-78	-36	119	-4.49
left posterior cerebellum					21	

BA: brodmann area; MNI coordinates: the coordinates of peak point located in the Montreal Neurological Institute space.

**Table 3:** Regions showing significantly higher ReHo in PSPD group than HC.

Regions	BA	MNI coordinates			Cluster	t
		X	Y	Z		
left middle frontal gyrus	10/46	-39	57	9	109	4.31
right superior frontal gyrus	8	24	27	48	356	6.33
right middle frontal gyrus	10				327	
right medial prefrontal cortex	8/9				152	
left superior frontal gyrus	8/6	-18	12	63	118	5.21
left medial prefrontal cortex	8				50	
right inferior parietal lobule	40/7	51	-42	39	84	5.36
left supramarginal gyrus	40	-54	-45	42	56	3.99

### Correlations between ReHo and VAS, SAS, SDS scores

**Fig 3:**
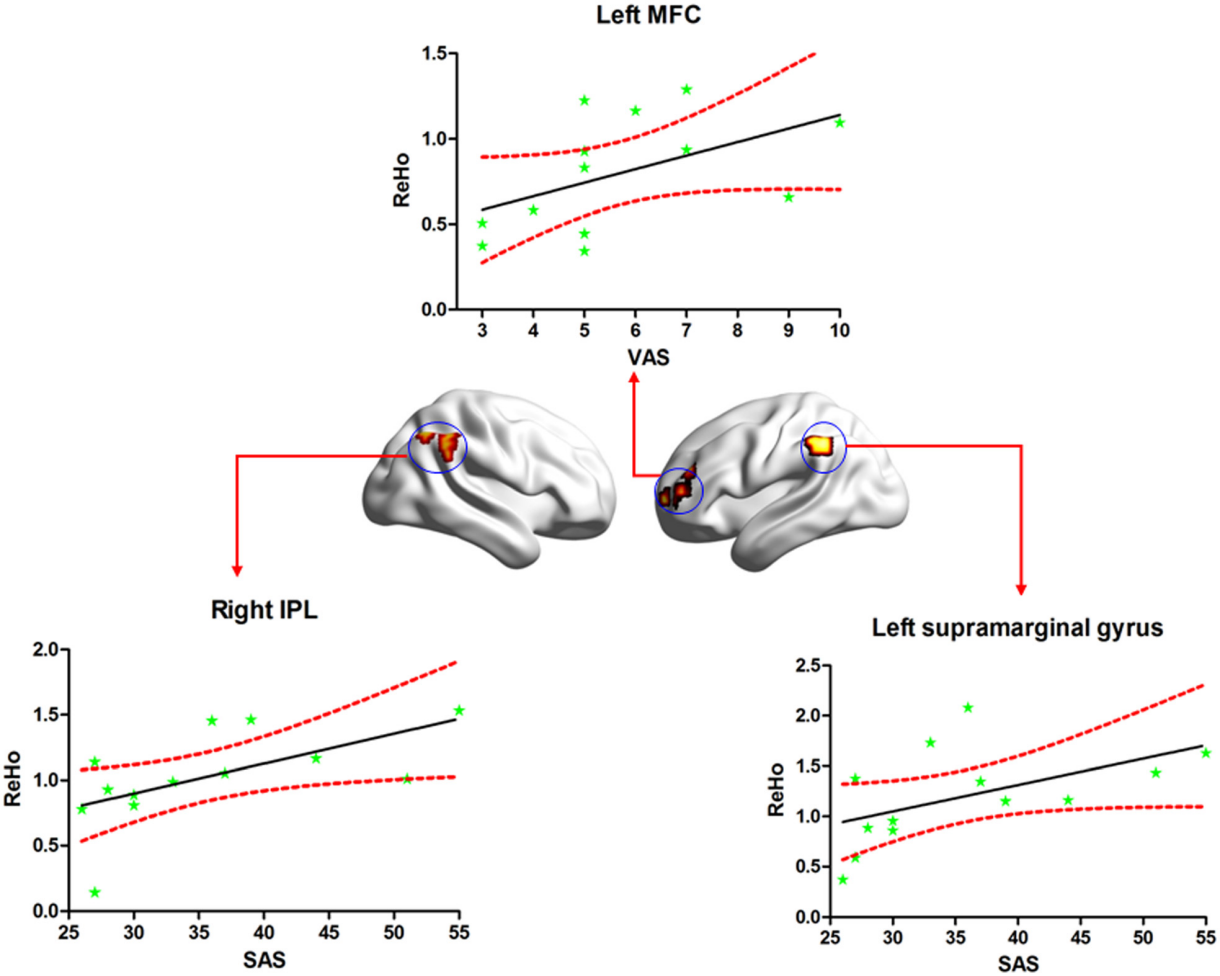
The clusters (blue circles) were associated with significant differences between the PSPD and HC groups, and differences within which the ReHo index (normalized ReHo value) significantly correlated with VAS and SAS. MFG: middle frontal gyrus; IPL: inferior parietal lobule; ReHo: regional homogeneity; VAS: Visual Analogue Scale; SAS: Self-Rating Anxiety Scale.

The significantly (*p*< 0.05, uncorrected) positive correlations were found between mean ReHo of the left MFG and VAS scores, and between mean ReHo of both left supramarginal gyrus and right IPL and SAS scores. The r values were 0.595 (*p =* 0.032,), 0.614 (*p =* 0.025), and 0.736 (*p =* 0.004), respectively (Fig 3). The significant correlation between duration of illness and mean ReHo was not found. In addition, the results of correlation analysis without removing the effect of age, gender and head motion were showed in S1 Fig. Furthermore, the ROC analysis was performed for these three regions. The results showed that the areas under the curves of left MFC, left supramarginal gyrus and right IPL were 0.906 (*p <*.001), 0.803 (*p* = 0.003), and 0.903 (*p <*.001), respectively, which implied that ReHo values of these three regions might be capable of differentiate the two groups. Then, the cut-off value and corresponding sensitivity and specificity of each brain region were further analyzed, and the results were displayed in S2 Fig and S1 Table.

### Reproducibility

**Fig 4:**
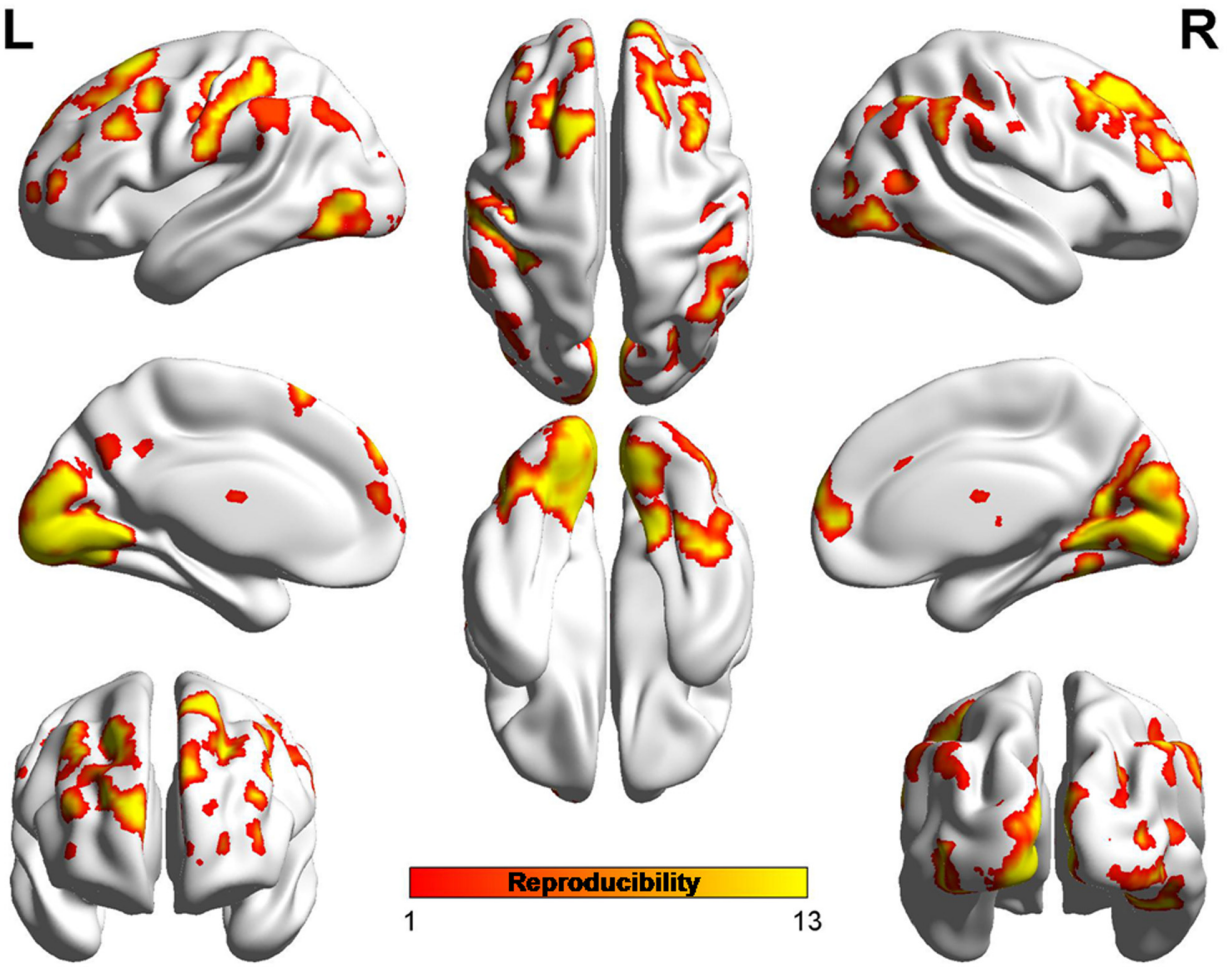
Leave-one-out sample validation. The group comparisons based upon the permutated samples (i.e., 12 PSPD vs. 23 HC) for total 13 times. For each voxel, the color indicates number of tests where this voxel exhibited significant group differences across the total 13 tests (i.e., the reproducibility).

The sample size was relatively small in the present study. To validate the reproducibility and robustness of these findings on ReHo, a leave-one-out validation was performed. This method has been used in previous study [40]. Specifically, one PSPD patient was left out of the sample, and the group comparisons were performed based upon the permutated sample (i.e., 12 PSPD vs. 23 HC). This led to total 13 two-sample t-test images, based on which, for each voxel, the number of tests where this voxel exhibited significant differences between groups across 13 tests was calculated as the reproducibility of the ReHo differences between PSPD patients and HC. Fig 4 indicated the highly reproducible patterns of ReHo changes across these tests as well as the original test reported. The results without removing the effect of age, gender and head motion were displayed in S3 Fig.

## Discussion

The results in present study supported our hypothesis, namely, that PSPD patients exhibit ReHo change in a number of regions related to pain compared with participants in the HC group. More importantly, we found the significantly positive correlations between the mean ReHo of the left MFG and VAS, and between the mean ReHo of both right IPL and left supramarginal gyrus and SAS scores. These results indicate that the altered ReHo in specific regions may be associated with the pathomechanisms underlying PSPD.

### Decreased ReHo values in PSPD patients

In the present study, significantly decreased ReHo was displayed in the bilateral S1, posterior cerebellum and occipital lobe. The S1 cortex, locating in the postcentral gyrus of the parietal lobe, is an important region of the lateral pain system and processes inputs related to the sensory system of touch, nociception, temperature, and proprioception [41]. Several studies have documented activation in the S1 cortex in response to painful stimulation using fMRI and PET in humans [42–45], and this activation involved the encoding of sensory discriminative aspects of pain, such as location, intensity and duration [46]. Previous studies have also found reduced ReHo and cortical activity in the S1 cortex using resting-state fMRI and EEG in chronic pain patients compared to HC subjects [10, 30]. In accordance with these findings, the decreased ReHo of the S1 cortex in present study may suggest dysfunction in the brain’s ability to efficiently discriminate the sensory aspects of pain in patients with PSPD. The cerebellum is primarily considered to be associated with skilled motor behaviors [47]. However, increasing evidence indicate that the cerebellum plays an important role in pain regulation (for review see [48]). Helmchen et al.(2003) firstly demonstrated that different parts of the cerebellum had distinct responses to perceived pain intensity during noxious thermal stimuli in healthy subjects [49]. A patient study showed that, compared with HC, the cerebellar areas related to cognitive processing were both activated by painful heat and painful brushing in chronic neuropathic pain patients. This suggests that cognitive processing areas in the cerebellum may be related to the encoding of pain [48]. In addition, the activation of cerebellum is also observed in studies on pain empathy [50], emotion [51] and pain anticipation processing [52]. Both functional and structural abnormalities of the cerebellum have been demonstrated in emotional disorders, including depression and schizophrenia [53]. Hence, the diminished ReHo of the posterior cerebellum in our PSPD group could imply a reduced modulation ability in pain multi-dimensional aspects, such as the coding of pain, negative emotions and anticipation of pain, and might be associated with more uncomfortable feelings related to pain and emotion than healthy subjects.

Additionally, although occipital lobe is traditionally thought to be involved in visual information processing [54], a recent rat study found that the occipital cortex was connected to some structures involved in pain-descending inhibitory mechanisms [55]. Several functional studies in humans have documented decreased signals in the occipital lobes of patients with chronic pain disorder relative to HC using different neuroimaging methods, including PET, EEG and resting-state fMRI [10, 56, 57]. Also, lesions in the occipital lobe result in visual disturbance, memory deficits and motion perception disorders [58]. Studies in migraine patients showed that the decreased ReHo in the occipital lobe negatively correlated with the disease duration [58], and gradually altered BOLD signals in this region, similar to the process of cortical spreading depression, was related to the pathogenesis of migraine aura [59]. In the present study, the reduced ReHo was also observed in the occipital lobe. Based on the results from previous studies on pain, we speculated that this finding might be related to the pathological mechanism underlying the dysfunction of sensation and cognition in PSPD patients. Future studies will be required to explore the relationship between the abnormal activation in occipital lobe and pain-related dysfunction with large sample size, which may provide more mechanistic explanations in humans.

### Increased ReHo values in PSPD patients

The PFC is an area of the forebrain located in the anterior part of the frontal lobes. This area is associated with thought orchestration and action in accordance with internal goals [60]. Previous studies have documented involvement of the PFC in the affective-motivational component of the pain experience as a part of the medial pain system [61, 62]. Increased prefrontal activation has also been found in chronic pain states such as sympathetically mediated and neuropathic pain and capsaicin-induced secondary hyperalgesia [63–65]. In a prior PET study, patients with painful mononeuropathy exhibited increased regional cerebral blood flow (rCBF) in the PFC during habitual pain states relative to alleviated states [66]. A recent fMRI study also showed enhanced activation of the PFC in response to pain stimulation in PSPD patients as compared to that observed in controls [32]. In line with these findings, the present study also showed increased ReHo in the PFC (including bilateral SFG, MFG and left IFG) in PSPD patients. Therefore, this result suggests that the PFC plays an important role in high-level pain modulation, and the increased ReHo of the PFC may be associated with the cognitive impairment observed in PSPD patients [5]. Moreover, a significantly positive correlation was found between the mean ReHo of left MFG and VAS scores in PSPD group. It is well known that the higher VAS scores, the worse pain. This positive correlation further manifested that the modulation of ReHo of MFC on the pain in PSPD patients is synchronous, which means a higher ReHo value corresponds to more severe pain impairment.

Increased ReHo values were also found in the DMN (including the mPFC, right IPL, and left supramarginal gyrus) in the PSPD group of the present study. The DMN regions are typically characterized by negative activation during task-fMRI, mainly involving the integration of cognitive and emotional processing and monitoring the surrounding environment [67]. Different parts of the DMN play distinct roles in the generation and transmission of pain. For example, mPFC is associated with the processing of emotional information and mediates the functional interactions among the brain regions that participate in pain processing, whereas precuneus is likely involved in the shifting of attention between different spatial locations [30]. Hence, the ReHo changes in regions of DMN might indicate the processing of pain involving emotionally intense information. Recently, a resting-state fMRI study demonstrated increased ReHo values exhibited in the DMN regions of healthy subjects at pain status as compared to the normal state [12]. Another research on pain stimulation, in the patients with cluster headache, indicated that the state of headache attack showed higher ReHo values in PCC and mPFC compared with those during out of attack [59]. Additionally, several recent pain-related studies showed an enhanced ReHo in some regions of DMN in patients group relative to HC [30, 68]. These results are consistent with those of the present study. The abnormal temporal coherence in DMN among patients suffering from chronic pain may be associated with the pathomechanism underlying the diseases. Furthermore, we also found significantly positive correlations between the ReHo of both right IPL (BA7) and left supramarginal gyrus and SAS scores. Previous studies with human samples as well as animal studies involving monkeys have demonstrated that the posterior regions (BA7) of the parietal cortex are associated with perceived pain and may reflect the time properties of pain perception [69, 70]. Given that several studies have reported the impaired emotion in patients with PSPD [2–4], the positive correlation may indicate that IPL and supramarginal gyrus are critical region related to the potential mechanism of the impaired emotional awareness in PSPD patients.

It is worth noting that ReHo is not merely a reliable metric to investigate the spontaneous neuronal activities in resting-state fMRI, but also has its neurobiological importance demonstrated by a recent study with large sample size [22]. The investigators revealed the gradient distribution of the functional homogeneity across the cortical mantle by examining the regional variation of ReHo in the whole brain and suggested that this measure most likely reflected the degree of regional segregation of brain function in humans. Moreover, the module organization was uncovered by the functional covariance network derived using ReHo, and this might indicate the integrated information processes related to high-order cognition of the human brain. These findings assign ReHo possible biological significance as a functional measure of regional segregation and integration of information processing. Thus, the altered ReHo of special regions in PSPD patients in present study, implies that the dysfunctions are associated with the disruption of complex information processing (perception of pain and modulation of emotion) in the brain. Additionally, strong correlations have been revealed between ReHo and morphological measures (e.g., cortical thickness, surface area, mean curvature, sulcal depth, and gyrification index) [22], although the abnormal brain structure were not found by conventional MRI in PSPD patients. Therefore, this relationship may suggest the structural basis of the change in ReHo and reflect the aberrant functional impairment accompanying morphological alterations in PSPD patients in present study, which prompts us to further explore the neural mechanism of PSPD by detecting the morphological metrics in those regions related to disease in future.

The present study uncovered abnormal spontaneous neuronal activities in some regions related to pain in PSPD using the ReHo method; nevertheless, some limitations still remain. First, the sample size was relatively small, and thus further studies with larger sample sizes are encouraged. Second, our exclusion criteria did not include treatments that might influence patients' pain perception[9], such as antidepressants, and this may bias the results of the present study.

## Conclusions

Our results document alterations of ReHo in the brain regions related to pain in patients with PSPD during a resting state. The disruptions of ReHo presumably induced by PSPD in the current study may be reflective of the abnormal spontaneous neuronal activities associated with persistent pain. We speculate that these ReHo changes are responsible for the observed deficits of sensation and affection in PSPD patients. Furthermore, our results also show that ReHo may be capable of characterize spontaneous brain dysfunction in PSPD. We hope that our findings will be helpful in improving the understanding of the neural mechanism underlying PSPD.

## Supporting Information

S1 Fig The correlation analysis without removing the effect of age, gender and head motions. The clusters (blue circles) were associated with significant differences between the PSPD and HC groups, and differences within which the ReHo index (normalized ReHo value) significantly correlated with VAS, SAS and SDS scores. MFG: middle frontal gyrus; IPL: inferior parietal lobule; ReHo: regional homogeneity; VAS: Visual Analogue Scale; SAS: Self-Rating Anxiety Scale; SDS: Self-Rating Depression Scale.
(TIF)

S2 Fig The ROC results showing that brain regions can significantly identify PSPD patients with relatively high sensitive and specificity. MFG: middle frontal gyrus; IPL: inferior parietal lobule; SG: supramarginal gyrus.
(TIF)

S3 Fig Leave-one-out sample validation without removing the effect of age, gender and head motions. The group comparisons based upon the permutated samples (i.e., 12 PSPD vs. 23 HC) for total 13 times. For each voxel, the color indicates number of tests where this voxel exhibited significant group differences across the total 13 tests (i.e., the reproducibility).
(TIF)

S1 Table ROC analysis for differentiating patients from healthy controls. MFG_L: left middle frontal gyrus; SG_L: left supramarginal gyrus IPL_R: right inferior parietal lobule; AUC: areas under the curves; cut-off value: the optimal value discriminated PSPD with HC; p: statistical p value. p < 0.05 represent that ReHo value of the regions has significantly diagnostic effect on PSPD, more bigger the AUC is, more effective the diagnostic value is.
(DOCX)
