# Supplementary figures and images for: Altered Spontaneous Activity in Patients with Persistent Somatoform Pain Disorder Revealed by Regional Homogeneity
Source: PLoS One. 2016 Mar 15;11(3):e0151360. doi: 10.1371/journal.pone.0151360 (PMC4792417; doi:10.1371/journal.pone.0151360)

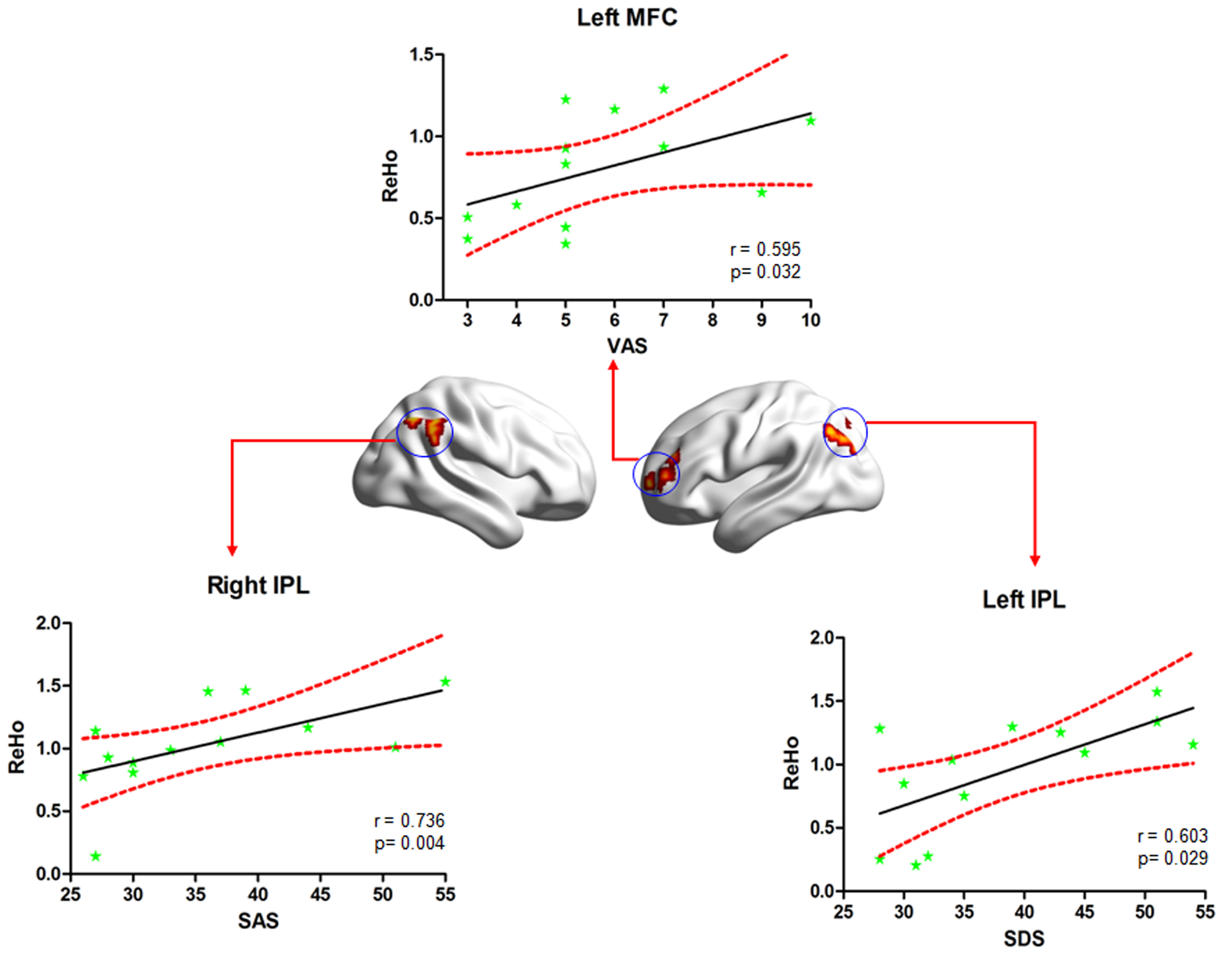

Supplement: S1 Fig — MFG: middle frontal gyrus; IPL: inferior parietal lobule; ReHo: regional homogeneity; VAS: Visual Analogue Scale; SAS: Self-Rating Anxiety Scale; SDS: Self-Rating Depression Scale. (TIF) [file pone.0151360.s001.tif]

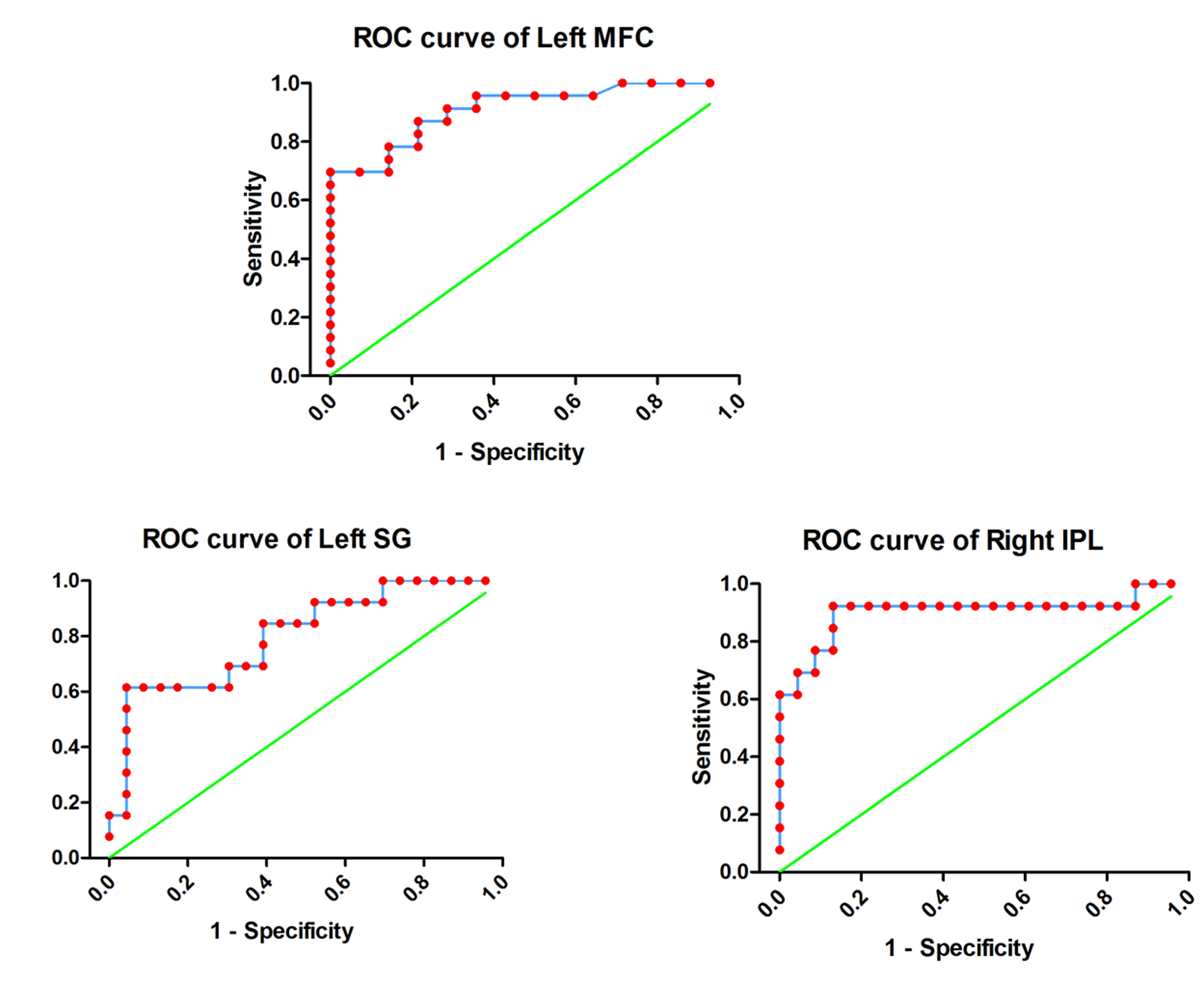

Supplement: S2 Fig — MFG: middle frontal gyrus; IPL: inferior parietal lobule; SG: supramarginal gyrus. (TIF) [file pone.0151360.s002.tif]

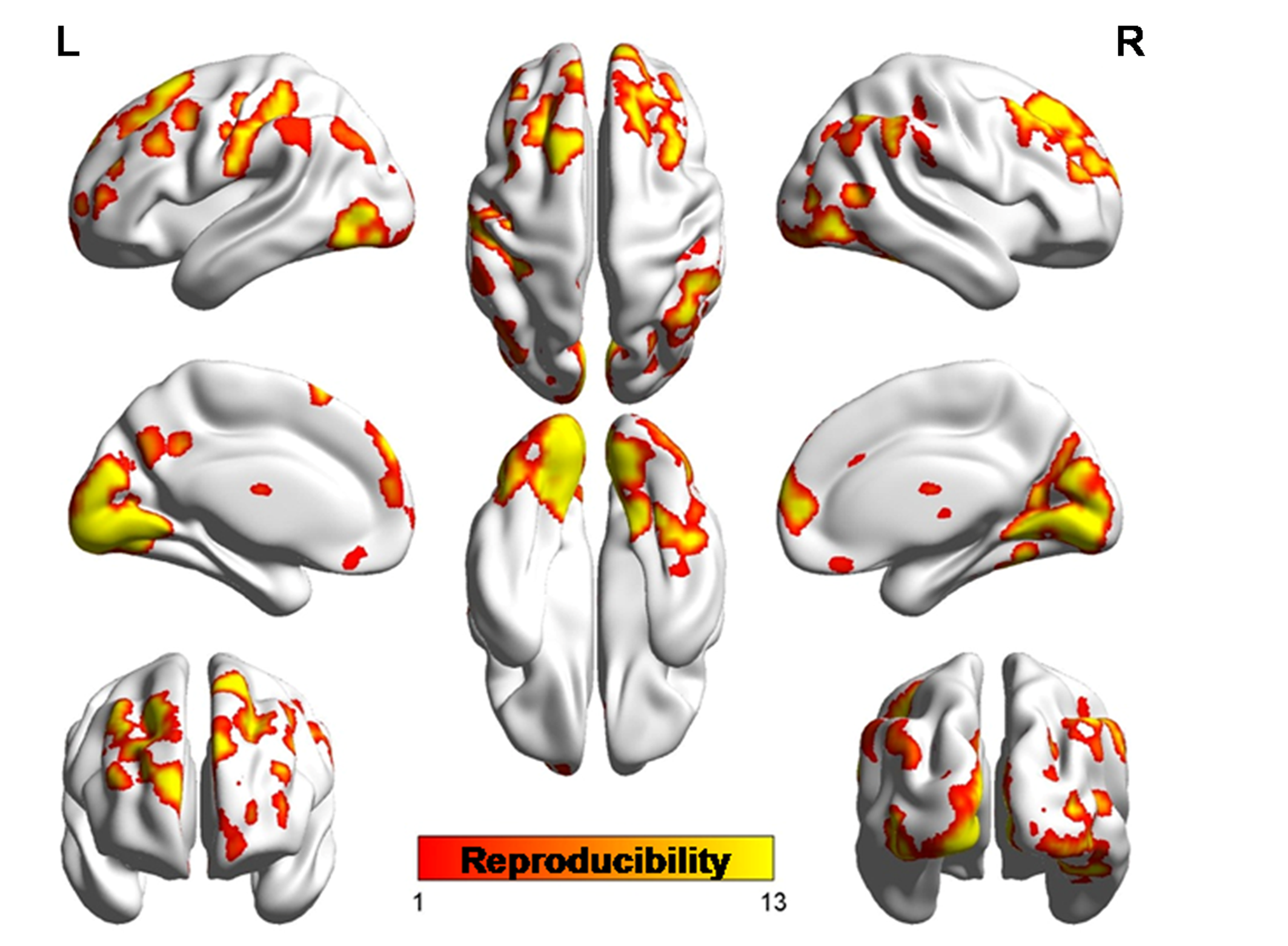

Supplement: S3 Fig — The group comparisons based upon the permutated samples (i.e., 12 PSPD vs. 23 HC) for total 13 times. For each voxel, the color indicates number of tests where this voxel exhibited significant group differences across the total 13 tests (i.e., the reproducibility). (TIF) [file pone.0151360.s003.tif]
