# Supplementary material for: Altered Spontaneous Activity in Patients with Persistent Somatoform Pain Disorder Revealed by Regional Homogeneity
Source: PLoS One. 2016 Mar 15;11(3):e0151360. doi: 10.1371/journal.pone.0151360 (PMC4792417; doi:10.1371/journal.pone.0151360)
Supplement: S1 Table — MFG_L: left middle frontal gyrus; SG_L: left supramarginal gyrus IPL_R: right inferior parietal lobule; AUC: areas under the curves; cut-off value: the optimal value discriminated PSPD with HC; p: statistical p value. p < 0.05 represent that ReHo value of the regions has significantly diagnostic effect on PSPD, more bigger the AUC is, more effective the diagnostic value is. (DOCX) [file pone.0151360.s004.docx]

| Brain regions | Sensitivity | Specificity | AUC | cut-off value | P |
| --- | --- | --- | --- | --- | --- |
| SG_L | 0.846 | 0.619 | 0.803 | 0.847 | 0.003 |
| IPL_R | 0.932 | 0.870 | 0.903 | 0.756 | 0.000 |
| MFG_L | 0.846 | 0.783 | 0.906 | 0.435 | 0.000 |
